# Local control on precipitation in a fully coupled climate-hydrology model

**DOI:** 10.1038/srep22927

**Published:** 2016-03-10

**Authors:** Morten A. D. Larsen, Jens H. Christensen, Martin Drews, Michael B. Butts, Jens C. Refsgaard

**Affiliations:** 1 Technical University of Denmark, Produktionstorvet, building 426, 2800 Kgs. Lyngby, Denmark; 2 Danish Meteorological Institute, Lyngbyvej 100, 2100 Copenhagen, Denmark; 3 DHI, Agern Alle 5, 2970 Hørsholm, Denmark; 4 Geological Survey of Denmark and Greenland, Øster Voldgade 10, 1350 Copenhagen, Denmark

## Abstract

The ability to simulate regional precipitation realistically by climate models is essential to understand and adapt to climate change. Due to the complexity of associated processes, particularly at unresolved temporal and spatial scales this continues to be a major challenge. As a result, climate simulations of precipitation often exhibit substantial biases that affect the reliability of future projections. Here we demonstrate how a regional climate model (RCM) coupled to a distributed hydrological catchment model that fully integrates water and energy fluxes between the subsurface, land surface, plant cover and the atmosphere, enables a realistic representation of local precipitation. Substantial improvements in simulated precipitation dynamics on seasonal and longer time scales is seen for a simulation period of six years and can be attributed to a more complete treatment of hydrological sub-surface processes including groundwater and moisture feedback. A high degree of local influence on the atmosphere suggests that coupled climate-hydrology models have a potential for improving climate projections and the results further indicate a diminished need for bias correction in climate-hydrology impact studies.

The critical importance of realistically representing the complex interactions and hydrological feedback loops between the atmosphere and the land surface in climate models is well documented^1^. The ability to adequately simulate precipitation systems is often related to the horizontal grid scales resolved by the models^2^ and their ability to account for non-linearity and the associated variability^3^,^4^. Also, this is highly dependent on a proper land surface representation as soil moisture acts as a control on water and energy fluxes from land surfaces to the atmosphere while humidity, temperature, wind, radiation and precipitation provides the drivers and input for the land-surface and subsurface water fluxes. In particular, on local to regional scales the models’ ability to represent a wide range of complex physical processes at a very high spatial and temporal resolution is crucial for describing the spatio-temporal variability of precipitation^1^,^5^,^6^. Some improvements in modelling approaches and improved confidence in precipitation projections have been seen recently. These improvements include: (I) higher resolution spatio-temporal model scales with grid resolutions down to e.g. 1.5 km^7^, (II) improved physical schemes and parameterizations (including dynamical vegetation schemes^8^) in climate models and the development of earth system models (ESMs)^9^, (III) better quality of forcing and evaluation data like the increased availability of high quality gridded data sets^10^,^11^ and (IV) a continuing growth of computational power. That said even state-of-the-art regional climate models (RCMs), which operate at scales down to a few kilometres, still generally suffer from deficiencies in fully representing the hydrological feedback to the atmosphere from the land surface and sub-surface^12^,^13^. Excluding groundwater in an RCM for example, prevents access to a possible moisture source for the atmosphere and additional surface cooling through evaporation under otherwise dry conditions. In some cases, this can degrade the land surface signal to the atmosphere^14^ and therefore the RCM performance, causing substantial biases under specific conditions^15^.

One approach to improve the formulation of RCMs is to employ a full dynamical coupling to land surface processes from a comprehensive 3D hydrological model that represents these processes at a higher spatio-temporal resolution using more complete physically based process descriptions. Such a coupling is essentially an analogue to the coupling between atmosphere and ocean models, which is currently considered the norm by all major global climate modelling groups. Only a few research teams have attempted this^16^,^17^,^18^,^19^ focusing mainly on shorter time scales in the range of days to weeks or by using land surface models excluding 3D subsurface flow. Implementing a dynamical coupling is not only a technical challenge^20^, but also poses additional problems since complex 3D hydrological models are typically adjusted to perform well at the catchment scale, but not for any arbitrarily large domain as typically specified for an RCM. The standard parameter adjustments obtained by matching (calibrating) the hydrological model to observations are lost when coupling to a climate model. The hydrological model is simply assigned to input from the atmospheric model to which it is not calibrated.

Here for the first time, we report on a multi-year experiment using a model setup^20^,^21^ where an RCM has been fully coupled to a detailed high resolution 3D hydrological model. It is at the longer time scales from seasons to years that the major impacts on model performance are expected and should be assessed. This is particularly the case for groundwater systems, with longer response times to changes in rainfall. The RCM-hydrology model coupling in the present study was performed over the Skjern River hydrological catchment in western Denmark (Fig. 1a) situated inside a larger RCM model domain. For the study area, groundwater plays an important role in the terrestrial water balance with a high degree of contact between aquifers and the vegetation root zone.

## Results

**Figure 1:**
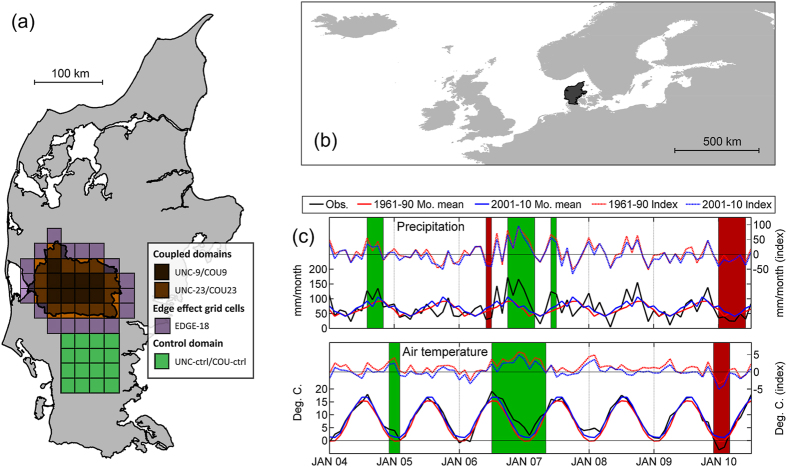
Hydrological catchment, regional climate model domain and observed precipitation and temperature during the study period. (**a**) Location map of the Skjern River hydrological catchment on the Jutland peninsula. The grids show the outline of the two RCM evaluation domains of 9 and 23 grid cells (UNC-9/COU-9 and UNC-23/COU-23) in the coupling domain (representing either a 100% overlap or grid cells with at least a 50% overlap respectively), the 16 grid cell RCM control domain (UNC-ctrl/COU-ctrl) and the 18 grid cell edge effect area (EDGE-18). (**b**) The RCM domain, with the Jutland peninsula highlighted. (**c**) The observed monthly precipitation and temperature for the simulation period 2004–2010, 1961–90 and 2001–10 mean values and index values (mean values subtracted from observations). The periods of high or low levels are outlined in green and red respectively for both precipitation and temperature. The plots were created using MATLAB (v. R2013A - www.mathworks.com) and QGIS (v. 2.4.0 - www.qgis.org) software.

**Figure 2:**
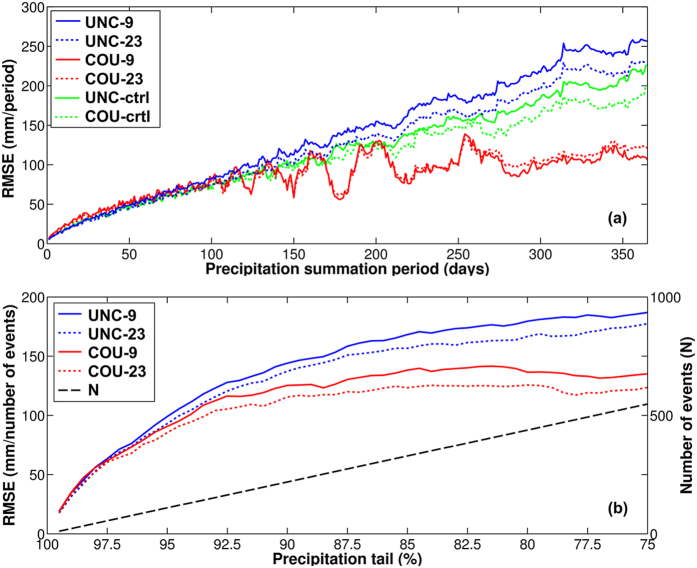
Simulation root mean square error as a function of period length and precipitation intensity. (**a**) Root mean square errors (RMSE) of observed and simulated precipitation after summation in periods of 1–365 days for the entire data range and (**b**) RMSE as a function of the precipitation sum from the 75–99.5% percentile (sorted by observation data) as well as the sample size (N).

**Figure 3:**
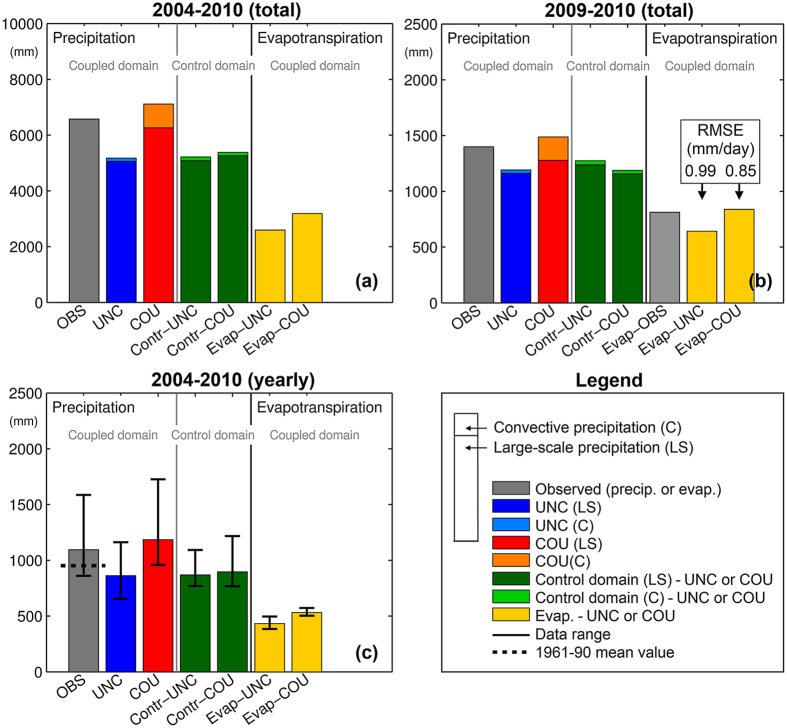
Precipitation and evapotranspiration amounts and variability. Plots of the observed total precipitation and simulated large-scale (LS) and convective (C) precipitation (stacked to show simulated totals) as well as the simulated and observed (2009–2010 only) evapotranspiration. Results are shown as summation plots for (**a**) the total period, (**b**) for the 2009–2010 period and (**c**) yearly averages (Aug to Jul). Plot (**b**) includes observed evapotranspiration and corresponding RMSE values for COU and UNC daily evapotranspiration. Plot (**c**) includes the total observation and simulation range for the six years and the 1961–90 mean (the gauge undercatch is corrected by monthly factors^48^). Over the coupling domain average levels from the 9 grid cells are shown (UNC-9 and COU-9) and precipitation results are shown for both the coupling domain and the control domain (see Fig. 1).

We examine here the results for the coupling domain, over the Skjern River catchment, simulated using both the original RCM (UNC) and the coupled climate-hydrological model (COU). Results covering a similar area but outside the coupling domain are also examined for both simulations (UNC-ctrl and COU-ctrl for the UNC and COU simulations respectively) (Fig. 1a) – (simulation and domain abbreviations can be found in Table 1 and the Methods section). Figure 2a shows the root mean square error (RMSE) between simulated and observed precipitation calculated for time intervals ranging from 1 to 365 days, over the entire simulation period of 6 years (2191 days). The simulations over the coupling domain (UNC and COU) and the control domain (UNC-ctrl/COU-ctrl) show similar precipitation RMSE levels for periods up to 100 days (seasonal) and the response is similar when comparing the 9 and 23 RCM grid cells (Fig. 1a). For periods exceeding 100 days, i.e. seasonal to annual, the COU simulation shows distinctly lower RMSE levels stabilizing at roughly 100–150 mm/period. This is compared to UNC levels of approx. 250 mm/period and UNC-ctrl and COU-ctrl levels of app. 200 mm/period. The magnitudes of single precipitation events strongly influence this RMSE analysis. Therefore the COU simulation results which exhibit more convective precipitation events (Fig. 3) show a correspondingly higher degree of fluctuations. Fig. 2b shows the RMSE in the upper 75–99.5% percentile and like Fig. 2a, the COU results are superior to the UNC results, for these heavy rainfall events.

Figure 3 shows the partitioning between large-scale and convective precipitation according to the parametrized processes of the RCM, the simulated precipitation amounts for the total period and annual average precipitation, together with the corresponding evapotranspiration amounts. The coupling is seen to have a substantial effect on the total precipitation amounts and the distribution between large-scale and convective precipitation over the coupling catchment domain, with a larger fraction of the convective precipitation. The total COU simulated precipitation over the entire simulation period is closer to the observations (OBS) than UNC (statistically significant at p = 1.44e-9, for the nine UNC-9/COU-9 grid cells). The COU simulation overestimates the total rainfall by 538 mm. In comparison, the UNC underestimates by 1402 mm. This corresponds to a reduction in absolute bias from 21% to 8% (Fig. 3a). The increase in precipitation from UNC to COU occurs both in the large-scale and convective precipitation contributions, which increase by 1204 mm and 626 mm respectively. The amount of convective precipitation increases by a factor of six. From 1 Jan. 2009, flux tower observations of evapotranspiration from catchment representative surfaces are available^22^. Despite inherent scale issues between point measurements and gridded model output, COU improves the simulated evapotranspiration amounts compared to UNC (Fig. 3b) (statistically significant at p = 1.65e-7, same grid cells as above). Hence, evapotranspiration simulated by COU (838 mm) is closer to the observed evapotranspiration (813 mm) compared to the evapotranspiration simulated by UNC (642 mm). The RMSE values are 0.85 and 0.99 mm/day for COU and UNC, respectively. Similarly, the intra-annual variability of total precipitation simulated by COU indicated by the error bars better resembles that of the OBS levels compared to UNC (Fig. 3c). It is also seen that, the maximum annual precipitation is notably higher for OBS and COU (1586 and 1726 mm) compared to UNC (1162 mm). The UNC and COU results in Fig. 3 are for the 9 grid cell coupling domain only, but the results are comparable whether assessed over the 9 fully coupled RCM grid cells or over the total 23 grid cells affected by the coupled land surface (see Fig. 2). In general, the control domain (Fig. 1a) shows precipitation levels comparable to the UNC run for both the UNC-ctrl and COU-ctrl runs (equal at a significance level of p = 0.07, same grid cells as above).

**Figure 4:**
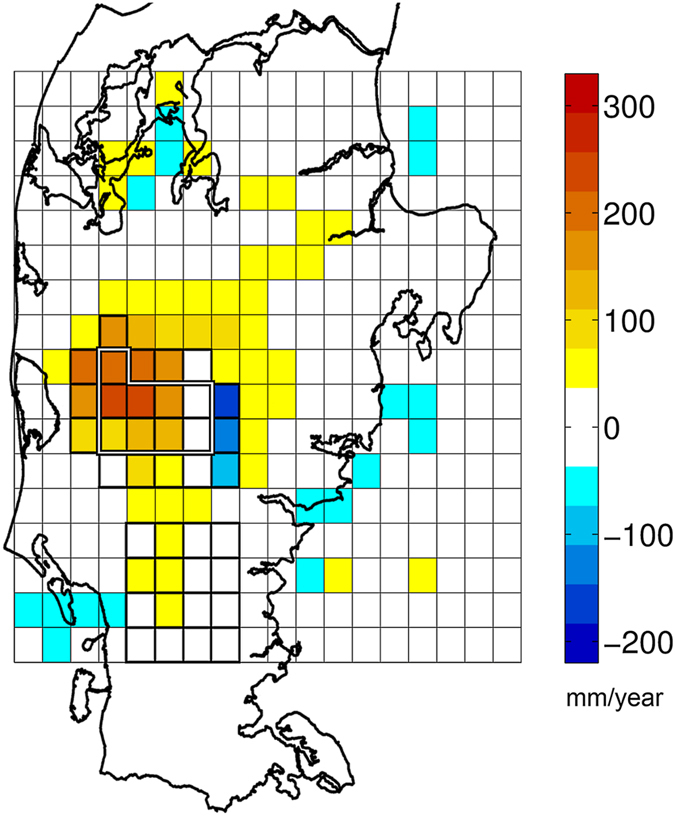
Distributed plot of the root mean square error difference between coupled and uncoupled simulations. Distributed plot of the difference (COU subtracted from UNC) in RMSE levels for a 365 day summation period over the entire Jutland peninsula. The colour scale intervals and the white colour (centred on 0 mm/year) reflects the internal model variability (+/−36 mm/year), as described in the method sections. The 9 and 23 grid cell coupling domains and the 16 grid cell control domain are outlined. The plot was created using MATLAB (v. R2013A - www.mathworks.com) and QGIS (v. 2.4.0 - www.qgis.org) software.

Figure 4 shows the spatial distribution of the differences between the coupled and the uncoupled models measured as the differences in RMSE levels for the annual (365 day) precipitation sum shown in Fig. 2. From this figure, we see that the stronger effects of the coupling are found within the coupling domain, although some deviations between UNC and COU can be seen outside this domain. For the white grid cells in Fig. 4, the differences are smaller than the estimated internal variability of the coupled model of 36 mm^21^.

## Discussion

The improved COU simulation results for time scales beyond 100 days, when compared to the UNC simulation results (Fig. 2a) implies that the more realistic treatment of subsurface processes becomes important when approaching the longer time scales typically simulated by climate models. Under Danish conditions with flat topography and a relatively large net precipitation, groundwater exerts a high degree of control on soil moisture conditions^23^. These subsurface processes are clearly better captured by the improved hydrology now incorporated into the climate model. This corresponds to previous findings^15^ arguing that soil moisture controls the ability of RCMs to reproduce the seasonal cycle for climates with characteristic summer and winter conditions. The present results also demonstrate the ability to improve the accuracy of simulated precipitation events in the upper tail. In particular, convective precipitation has been shown to be strongly influenced by soil moisture^24^ and the extreme tail of precipitation events includes a substantial fraction of convective precipitation^25^,^26^. In this study this is better resembled for the COU simulation. This implies that the improved hydrology included in the coupled RCM simulation is also important for the upper tail of precipitation events. Altogether the results imply that the need for bias correction in climate-hydrology studies using this coupled model may be substantially diminished or potentially eliminated. The comparable RMSE levels obtained for the UNC and COU simulations over the uncoupled control domain (UNC-ctrl and COU-ctrl respectively) indicate that the coupling only influences model results within or very close to the coupling domain.

The importance of soil moisture on the land surface-atmosphere interaction is primarily through its influence on the partitioning of incoming energy into sensible heat and latent heat (evapotranspiration) which affects atmospheric conditions in terms of temperature, stability and to some extent precipitation^1^. The substantial differences in precipitation between COU and UNC as discussed above arise in part from the increase in convective precipitation for the COU simulations. This may be explained by the relatively large differences in simulated evapotranspiration (Fig. 3a) over the coupling domain, confirming that a high degree of local scale influence of the precipitation is captured in the coupled model as also found previously for shorter periods^20^,^21^.

Over the coupling domain, the annual evapotranspiration (596 mm) in the COU simulations exceeds the corresponding UNC annual levels by approx. 100 mm/year, for the entire simulation period. For the evaluation period where detailed observations were available (from 1. Jan. 2009) the COU evapotranspiration was much more accurately estimated as indicated by the RMSE statistics (Fig. 3b). We attribute these results to the more realistic evapotranspiration scheme of the hydrological model and a better connection from the groundwater to the atmosphere. The UNC scheme is very simplistic with a single top soil layer only, resulting in drier soils during the summer season and a lack of soil moisture available for evapotranspiration. The COU scheme on the other hand is parameterized using multiple layers on a centimetre scale in the unsaturated zone, includes root zone/vegetation processes and most importantly allows the exchange between groundwater and surface layers. Prior to the coupling, rigorous calibration of the hydrological model was performed to make the model simulations match observed data on river discharge, evapotranspiration and groundwater head best possible and thus remove biases in model simulations of these fluxes and states^23^,^27^,^28^. The effect of the coupling is seen only for coupled grid cells and in the immediate vicinity (Fig. 4). Hence, we argue that the main share of differences between UNC-ctrl and COU-ctrl (Fig. 3a–c) are within the range of inherent model variability seen in RCMs^4^, including the RCM used in this study^21^.

The limited influence of the coupling on simulated precipitation beyond the coupling domain is also shown in Fig. 4. A statistical test shows that the impacts of the coupling for the nine fully coupled grid cells (COU-9) inside the edge of the coupling are significantly different from the impacts on 18 grid cells (EDGE-18, see Fig. 1) located just outside the edge of the coupling, indicating that the changes in the fully coupled domain are not caused by edge effects. Also, careful inspection of distributed energy fluxes along the edges revealed no impacts of numerical shocks caused by the model blending (not shown). In the immediate vicinity of the coupling domain there are small, but statistically significant, effects of the coupling. These differences can be explained by “downwind” propagation of the differences in the atmospheric variables. Biases near the land-sea contrast are due to deficiencies of the RCM and are similar to findings reported by other authors^29^,^30^.

With the present study, we demonstrate that convective precipitation is altered substantially by the coupling. Better access to subsurface moisture during the relatively dry but convectively dominated periods (mostly summer) enables increased moistening of the atmosphere. We note, however, that this will not necessarily hold true for convective parameterization schemes different from that used in the present RCM. It is not the intention of this work to test different convective parameterisations. Rather we want to emphasize that the coupling not only works, but that it shows a clearly improved performance and hence enhances the credibility of the RCM simulated climate over the Skjern River catchment. While one approach to improve the representation of land- and sub-surface processes in RCMs is to embed more advanced schemes directly, for example the WRF-Hydro model^31^, this paper aims to explore the full benefit of coupling to a detailed hydrological model with all the main components of the terrestrial water cycle including 3D groundwater flow.

Although the size of the domain where the full coupling is active is relatively modest in extent, it is large enough to allow for local feedbacks to act. The 2500 km^2^ river catchment corresponds to approximately 21 RCM grid cells which are directly affected by the coupling. Since current larger scale dynamic hydrological models of nationwide, regional or continental extent^32^,^33^ do not include full 3D detailed groundwater flow, and only rarely include energy based land surface fluxes, one perspective for this study is the potential for employing a dynamical model coupling over a considerably larger region. Evidently, such a study would contribute further to the assessment and validity of the newly developed coupled RCM-hydrology results presented here. Including these processes for the entire land domain of the RCM would constitute a considerable improvement and represent a great stride towards achieving regional ESMs. However, the spatial scale of both the domain (or catchment) size and the model resolution is obviously different between hydrological models representing 3D groundwater and most RCM (and ESM) models. Hence increasing the extent of the hydrology model without sacrificing detail would require an immense increase in the effort spent in the setup and calibration phase and the setup would require extensive ground truth data. Nevertheless, utilizing hydrological models at a wider extent, in a coupled model could be both feasible and achievable and would be able to provide an improved land surface representation in RCMs. In this respect, the present study with its demonstration of the significant impacts of such coupling is a major step towards such a goal.

## Summary and Conclusions

We have shown that the addition of comprehensive land surface and subsurface processes, from an advanced hydrology model, into an RCM, by using a fully dynamic two-way coupling substantially improves the RCMs’ simulation of precipitation. The improvements include both extreme and convective precipitation. This addresses, to some extent, the important challenge of producing realistic climate model representations of regional scale hydrology forcings and feedbacks under present-day climate conditions. Given the more physically based approach, this enables an increased confidence in RCM simulations of projected changes in the hydrological cycle. Our results clearly document the potential of including higher levels of spatio-temporal detail in the land-surface and subsurface using an external dynamically coupled hydrological land surface model. This is especially relevant for model studies of the impact of future climate change on the hydrological cycle and feedbacks to the climate system. This potential improvement however, cannot be achieved before it becomes possible to apply these high-resolution hydrological model setups to multiple adjacent hydrological catchments or preferably for the entire or at least a majority of the land area covered by the RCM. While this remains challenging in practical terms, we have indicated how some of the potential benefits may be realized.

## Methods

### Hydrological setup

The coupled setup uses the spatially distributed MIKE SHE hydrology model^34^ utilizing an integrated description of the following hydrological components: 1D Richards’ for the unsaturated zone, 3D groundwater flow with 11 vertical, geologically based, layers; 2D overland flow based on kinematic routing and 1D river routing. In addition, the model has been modified to include the SWET land surface model^20^ to accommodate energy based flux calculations from the two layers of soil and vegetation. The model has been widely applied for both research and applied management studies focusing on e.g. climate change effects on hydrology^35^, water management^36^, water quality^37^ and eco-hydrology^38^. The coupled hydrological catchment covers the 2500 km^2^ area of the Skjern River catchment located in the western part of the Jutland peninsula dominated by sandy soils and low topography (0–130 m.a.s.l.). The catchment model was initially extracted from the Danish national water resources model covering all of Denmark^39^ at 500 m resolution. The basic computational time step is 60 minutes. Automatic time step reduction is used for decreasing the maximum time step if precipitation exceeds 4 mm pr. time step. 36 parameters from the SWET land surface, river and unsaturated zone components were assessed for sensitivities against energy flux and discharge output measures whereas the saturated zone was already well calibrated^39^. The ten most sensitive parameters were calibrated against observations and the results were assessed for an independent validation period^28^.

### RCM setup

The climate model component used in the coupled setup is the state-of-the-art hydrostatic RCM HIRHAM version 5^ ^40 with an 11 km rectangular grid resolution. The model has been applied in numerous large scale multi-model studies such as PRUDENCE^41^, ENSEMBLES^42^ and CORDEX^43^ and it has been further developed with a focus on Northern Europe to include an ocean component^44^ and here an interactive hydrological model^21^ which is still a unique feature^20^. Furthermore, it has been used extensively at high resolution over Greenland^45^. The RCM domain covers app. 4000 km × 2800 km, the model is forced by ERA-Interim re-analysis data^46^ and an internal time step of 90 s is used. The optimal domain resolution and location characteristics were chosen from eight test domains^30^. The physical parameterization of precipitation in HIRHAM version 5 uses two separate precipitation schemes: A stratiform cloud condensation scheme for large-scale precipitation and a cumulus convection scheme for convective precipitation.

### Coupling

The coupled setup operates across platforms – MIKE SHE operates on a Windows PC, whereas HIRHAM is Linux based and operated on a parallelized CRAY XT5 supercomputer^20^,^21^. Both components were modified for compatibility with the OpenMI^47^ open source coupling protocol. OpenMI facilitates timing, spatial mapping, unit conversions and interpolation or summation for each time step. The data transfer between HIRHAM and MIKE SHE occurs at 30 min time steps, which was found to be an optimal trade-off between performance and computation^21^. The exchange variables from HIRHAM to MIKE SHE include the six driving climatic variables; precipitation, surface temperature, surface pressure, relative humidity, global radiation and wind speed. MIKE SHE in return delivers sensible heat (recalculated from surface temperature) and latent heat^20^. In this manner, the original HIRHAM land surface scheme is directly replaced by MIKE SHE within the coupling domain. Thus, the HIRHAM atmospheric fields are calculated based on the MIKE SHE land surface scheme. Outside the shared domain, HIRHAM applies its usual land surface scheme. The MIKE SHE fluxes are spatially aggregated using a weighted mean to match the HIRHAM grid cells.

### Simulations

The coupled (COU) and uncoupled (UNC) simulations start on 1 May 2004. The first 3 months are treated as spin-up, so the analysis period covers the period from 1 August 2004 to 31 July 2010. The simulation includes a very wet late summer and fall (2004), a dry summer (2006), a wet and warm fall/winter (2006–07), a wet summer (2007) and a cold and dry winter, spring and early summer (2010) (Fig. 1). In summary, the simulated period contains several instances of both heavy rainfall events and dry spells that differ substantially from the monthly means of both the standard climatological reference period 1961–90 and the decade 2001–2010 (Fig. 1c). The hydrological model uses an offline spin-up, continuously looping over a 3-year simulation period until reaching steady state on 1 May 2004.

### Analysis

The coupled simulation (COU) over the Skjern River catchment is compared to a simulation from a standard (uncoupled) RCM configuration (UNC) which does not include the improved hydrology component (Table 1). Output from the RCM is summarized to daily values corresponding to the time resolution of the observations. The observed precipitation data are gridded (10 km) and have undergone quality control. The observations have been dynamically corrected for gauge undercatch, based on daily gridded temperature and wind observations^27^ rather than using uncorrected or monthly mean corrected precipitation^48^. Bi-linear interpolation in space has been employed to fit the grid cells of the simulation enabling analyses in equal spatial and temporal resolution. We evaluate results from the RCM grid cells with a 100% overlap with the domain of the hydrological model (9 RCM grid cells entitled UNC-9 or COU-9) and a larger domain also including grid cells with a 50–100% overlap (23 RCM grid cells entitled UNC-23 or COU-23), (Fig. 1a). UNC and COU simulation results are also compared to results in a domain adjoining the coupling domain. This additional “control” domain (16 grid cells) is included in the analysis (Fig. 1a) by extracting results over the control domain from the UNC and COU simulations (Table 1). No overlap between the control domain and the coupling climate-hydrology domain is present and therefore the standard RCM land surface scheme is being applied within the control domain for both the UNC and COU simulations. Control domain results are entitled UNC-ctrl and COU-ctrl, respectively. The control domain is comparable in size to the coupling domain and represents hydrological conditions that are similar to the Skjern River. Furthermore, we tested for potential model deficiencies due to both grid cell blending and shock effects along the edge of the coupling domain: Assuming similar edge effects inside and outside the edge, the nine fully coupled grid cells (UNC-9/COU-9) are compared to the 18 grid cells (EDGE-18) located outside the edge (Fig. 1a). Hence, a two-sample two-tailed t-test of the RMSE differences between the UNC and COU simulations for these two groups of grid cells was performed employing quantile plots to test for normality (n = 9 and 18, α = 0.05). To reject the hypothesis that the RMSE differences in the nine fully coupled grid cells are caused by edge effects, the means of these two groups, allowing different variances, should be statistically non-identical.

The analysis on model performance for different period lengths (Fig. 2a) is computed as the root mean square error (RMSE) between observations and simulated variables over intervals ranging from 1 to 365 days using data from UNC-9/COU-9 and UNC-23/COU-23. The analysis of heavy precipitation events (Fig. 2b) shows the RMSE as a function of the number of precipitation events from the 75–99.5% percentiles corresponding to the largest 1–548 events. Observed and simulated precipitation (large-scale and convective) and evapotranspiration are plotted as totals for the 2004–2010 period (Fig. 3a) and the 2009–2010 period (Fig. 3b). Observed evapotranspiration is only available from 1 January 2009. Observed Skjern River catchment evapotranspiration is derived as surface area weighted values from three flux towers within the catchment that represent three different land use types. These three land use types represent 98% of the overall catchment land use. The statistical significance using a two-sample two-tailed t-test of the COU precipitation and evapotranspiration performance compared to UNC is assessed for the nine fully coupled grid cells employing quantile plots to test for normality (n = 9, α = 0.05). The internal model variability of the coupled model is calculated from eight perturbed one-year simulations^21^ using the same model setup and study area. From these simulations we estimated the standard deviation of the annual precipitation to 45 mm. We subsequently adjusted this to 18 mm for our case to reflect that the 365 day RMSE values are averages over a six year period. This resulted in a 95% confidence interval of [−36 mm; +36 mm] for the internal variability of 365 days precipitation. This yearly internal model variability is used as colorbar intervals in Fig. 4 showing the distributed difference in RMSE levels for each model grid cell (COU subtracted from UNC) for the 365 day summation period.

## Additional Information

**How to cite this article**: Larsen, M. A. D. *et al.* Local control on precipitation in a fully coupled climate-hydrology model. *Sci. Rep.* **6**, 22927; doi: 10.1038/srep22927 (2016).

## Figures and Tables

**Table 1:** Simulation specifications.

Simulation description	Output domains			
	Coupling domain		Control domain	
Default HIRHAM over the entire domain	UNC-9/UNC-23 (no coupling)	9 or 23 grid cell evaluation	UNC-ctrl (no coupling)	16 grid cell evaluation
HIRHAM coupled with MIKE SHE over the coupling domain. Default HIRHAM in remaining domain.	COU-9/COU-23 (coupled)	9 or 23 grid cell evaluation	COU-ctrl (no coupling)	16 grid cell evaluation

Coupled state, output evaluation domains, simulation and domain names and number of evaluation grid cells.
